# Use of medications by people with chronic fatigue syndrome and healthy persons: a population-based study of fatiguing illness in Georgia

**DOI:** 10.1186/1477-7525-7-67

**Published:** 2009-07-20

**Authors:** Roumiana S Boneva, Jin-Mann S Lin, Elizabeth M Maloney, James F Jones, William C Reeves

**Affiliations:** 1 Centers for Disease Control and Prevention, 1600 Clifton Road, Atlanta, Georgia 30333, USA

## Abstract

**Background:**

Chronic fatigue syndrome (CFS) is a debilitating condition of unknown etiology and no definitive pharmacotherapy. Patients are usually prescribed symptomatic treatment or self-medicate. We evaluated prescription and non-prescription drug use among persons with CFS in Georgia and compared it to that in non-fatigued *Well *controls and also to chronically *Unwell *individuals not fully meeting criteria for CFS.

**Methods:**

A population-based, case-control study. To identify persons with possible CFS-like illness and controls, we conducted a random-digit dialing telephone screening of 19,807 Georgia residents, followed by a detailed telephone interview of 5,630 to identify subjects with CFS-like illness, other chronically *Unwell*, and *Well *subjects. All those with CFS-like illness (n = 469), a random sample of chronically *Unwell *subjects (n = 505), and *Well *individuals (n = 641) who were age-, sex-, race-, and geographically matched to those with CFS-like illness were invited for a clinical evaluation and 783 participated (48% overall response rate). Clinical evaluation identified 113 persons with CFS, 264 *Unwell *subjects with insufficient symptoms for CFS (named ISF), and 124 *Well *controls; the remaining 280 subjects had exclusionary medical or psychiatric conditions, and 2 subjects could not be classified. Subjects were asked to bring all medications taken in the past 2 weeks to the clinic where a research nurse viewed and recorded the name and the dose of each medication.

**Results:**

More than 90% of persons with CFS used at least one drug or supplement within the preceding two weeks. Among users, people with CFS used an average of 5.8 drugs or supplements, compared to 4.1 by ISF and 3.7 by *Well *controls. Persons with CFS were significantly more likely to use antidepressants, sedatives, muscle relaxants, and anti-acids than either *Well *controls or the ISF group. In addition, persons with CFS were significantly more likely to use pain-relievers, anti-histamines and cold/sinus medications than were *Well *controls.

**Conclusion:**

Medical care providers of patients with chronic fatigue syndrome should be aware of polypharmacy as a problem in such patients, and the related potential iatrogenic effects and drug interactions.

## Background

Chronic fatigue syndrome (CFS) is diagnosed based on self-reported symptoms and exclusion of other illnesses that could cause the symptoms. There are no diagnostic clinical signs or laboratory markers for CFS. Thus, both health care providers and patients express concern about uncertainties in the diagnosis and management of the illness. This may be reflected in the apparent conundrum that persons with CFS have on average 22 healthcare visits per year [1] while only 20% of persons with CFS identified from the general population have been diagnosed with CFS [2,3].

Because the cause and pathogenesis of CFS remain inchoate, no definitive pharmacotherapy exists [4]. Many health care providers prescribe medications to treat the most bothersome symptoms – fatigue, muscle or joint pain, un-refreshing sleep and cognitive impairment. Most people with CFS who are under medical care have been ill for at least 5-years and may become frustrated with a lack of acceptable recovery. They often consult several providers and also self-medicate to treat their symptoms [5,6]. However, both prescribed and over the counter medications may cause untoward side effects, which may lead to new symptoms and exacerbate overall disability. We are aware of only one published population-based study (conducted in Wichita, Kansas) that documented medication use by persons suffering CFS and found that persons with CFS were more likely to use pain relievers, hormones, antidepressants, gastrointestinal and central nervous system medications [7]. We conducted the present analysis to critically evaluate use of prescription and non-prescription drugs (and supplements) by persons with CFS as compared to *Well *controls and persons who do not fully meet criteria for CFS (referred to as ISF). We used more recent data collected from defined metropolitan, urban, and rural populations in Georgia.

## Methods

### Study design

The study was approved by the Institutional Review Board of the Centers for Disease Control and Prevention and adhered to the human research guidelines of the U.S. Department of Health and Human Services. All participants were volunteers who gave informed consent.

**Figure 1:**
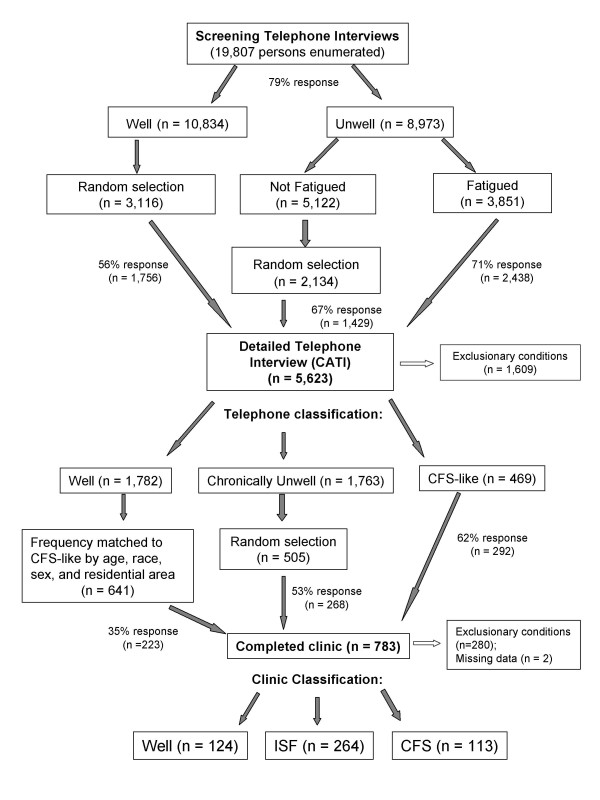
**Flow chart of subject sample derivation for the population-based, case-control study of chronic fatigue syndrome in Georgia, USA, 2004–2005**.

We conducted a population-based, case-control study to identify persons with CFS, *Unwell *and *Well *persons. Figure 1 represents a flow chart of how the subject sample was derived and details have been published earlier [8]. Briefly, between September 2004 and July 2005 we used random digit dialing to conduct a household ***screening ***interview with a household informant in three geographic areas in Georgia (metropolitan, urban and rural). The household informant described demographics and health status of household members 18 to 59 years old; that initial interview enumerated 19,807 adult residents and screened for unwellness among household members, based on having at least one CFS symptom (fatigue, impaired cognition, un-refreshing sleep, muscle or joint pain); *Well *residents had none of these symptoms for ≥ 1 month. The screening interview revealed 10,834 (55%) *Well *persons, 5,122 (26%) persons who were *Unwell *for at least a month but not fatigued, and 3,851 (19%) who were *Unwell and fatigued *for at least a month. We then conducted ***detailed ***telephone interviews with all those identified as *Unwell with fatigue*, a random selection of those who were *Unwell but without fatigue *and a random sample of *Well *persons (see Figure 1). Based on their responses to the detailed telephone interview, we classified participants as *CFS-like *if they met criteria of the 1994 CFS case definition [9]; as *chronically Unwell *if they endorsed some but not all CFS symptoms and as *Well *if they reported no such symptoms. Finally, we invited all 469 persons classified as CFS-like, 641 *Well *persons matched to the CFS-like by sex, race/ethnicity, age, and geographic stratum and a similar number (n = 505) of randomly selected *Unwell *persons for a one day clinical evaluation. Overall, 48.5% completed the clinical evaluation.

### Illness classification

To identify medical conditions considered exclusionary for CFS [9,10], the clinical evaluation included a standardized past medical history, a review of systems, a standardized physical examination, and routine laboratory testing of blood and urine. To identify psychiatric conditions considered exclusionary for CFS, licensed and specifically trained psychiatric interviewers administered the Structured Clinical Interview for DSM-IV (SCID) to diagnose Axis I psychiatric disorders and the Zung self-rating depression scale (SDS) to measure severity of depression [11]. Medical and psychiatric evaluations identified medical or psychiatric conditions considered exclusionary for CFS in 280 (36%) of the clinic participants; they and two others who had incomplete data were excluded from the analyses, leaving a total sample of 501 subjects for analyses.

We diagnosed CFS according to criteria of the 1994 case definition [9] and as recommended by the International CFS Study Group [10], which is standard in CDC studies of CFS [8,12]. Thus, we evaluated functional impairment by means of the Medical Outcomes Short-Form Health Survey (SF-36) [13]; we used the Multidimensional Fatigue Inventory (MFI-20) [14] to measure characteristics of fatigue and we utilized the CDC CFS Symptom Inventory to document occurrence, frequency and severity of the defining symptoms [15]. Subjects who had ≥ 4 case defining symptoms and exceeded the Symptom Inventory cut-off score, and met CFS cut-off scores on the SF-36 and the MFI-20, were considered to have CFS (n = 113 participants). Those who met at least one, but not all CFS criteria, comprised the ISF group (n = 264) and those who met none of the cut-off criteria comprised the *Well *group (n = 124).

### Data collection

We solicited demographic information during the detailed telephone interview and confirmed it at clinic. Clinic participants completed a battery of questionnaires prior to their clinic appointment, including questions concerning annual household income and health care utilization. In addition to completing questionnaires, we instructed participants to bring all medications (prescription and over the counter drugs and supplements) used within the past 2 weeks to their clinic appointment, where a nurse recorded the name, dose, reason and frequency of use. Information on reason for taking a medication was obtained primarily by general inquiry and recorded by clinical investigators using participant's or investigator's terminology of their own choosing.

For the purpose of this study we use the term "drugs" to refer to all prescription medicines and all non-prescription medicines that are available over the counter, but are not supplements or homeopathic medications. We use the term "supplements" to denote nutritional supplements, including vitamins, minerals, amino acids, fatty acids, homeopathic preparations and herbs.

A physician review panel from the CDC CFS Research Program reviewed the verbatim data recorded at clinic and verified names of drugs and supplements by means of the Physicians Desk Reference (PDR) or through website databases. The panel utilized generic name and ingredients to categorize individual drugs into 287 groups and an additional group for supplements. Based on their main effects, we grouped drugs into a smaller number of major categories. For the purpose of this study we kept the major drug categories similar to our previous study of drug use by persons with CFS [7]. The present analysis is limited to drugs used by at least 5 of the 501 subjects.

### Statistics

We used Chi-square (χ^2^) or Fisher's exact tests of independence to compare the distribution of categorical demographic characteristics by the three study groups and to assess differences in frequency of use of various medications by the three study groups. We used the Kruskal-Wallis test to compare differences in income, age and BMI by study groups. We used logistic regression to compute odds ratios (OR) for medication use in the CFS group relative to the ISF and *Well *groups; the Wald test was used to compute 95% confidence intervals as measures of the precision of the OR. We adjusted the analyses for potential confounders (household income, BMI, age, sex, race and geographic stratum) by including them as covariates in the regression models. The Hosmer-Lemeshow test served to assess the goodness of fit for multivariate logistic regression models.

## Results

### Descriptives and demographics

The CFS group was similar to the ISF and *Well *groups with respect to the distribution of age, sex, race and geographic stratum (Table 1). The *Well *group had a significantly higher household income (p < 0.001) and significantly lower BMI compared to the CFS and ISF groups (p < 0.01 for both).

**Table 1:** Basic demographic characteristics of the subjects with chronic fatigue syndrome (CFS), subjects with insufficient symptoms to be CFS (ISF) and *Well *controls

Demographic characteristic	CFS (n = 113)	ISF (n = 264)	Well (n = 124)	P
Race, n (%)				0.19
Caucasian	84 (74.3)	196 (74.2)	95 (76.6)	
Black	21 (18.6)	55 (20.8)	28 (22.6)	
All other	8 (7.1)	13 (4.9)	1 (0.81)	
Area, n (%)				0.98
Metro	23 (20.4)	54 (20.5)	22 (17.7)	
Urban	37 (32.7)	84 (31.8)	42 (33.9)	
Rural	53 (46.9)	126 (47.7)	60 (48.4)	
Female sex, n (%)	92 (81.4)	201 (76.1)	93 (75.0)	0.44
Age in years, mean (sd)	44.3 (10.1)	43.1 (10.4)	44.5 (10.5)	0.37
Median age	44.0	45.0	47.0	
Age range	18–59	18–59	19–59	
BMI, mean (sd)	27.5 (5.0)	27.5 (5.2)	26.0 (5.3)	0.018
Median BMI	27.0	27.0	25.0	
BMI range	17–39	16–39	18–38	
Income				0.017
Mean (sd)	64,495.8 (87,057.0)	67,455.6 (63,118.1)	85,599.2 (82,699.2)	
Median	52,025.0	55,000.0	72,272.0	
Income range	0.0 – 750,000.0	0.0 – 447,466.0	0.0 – 500,000.0	

BMI, Body mass index.

### Overall use of drugs and supplements

The 501 participants brought in 2,205 individual preparations that they were taking, of which we considered 1,557 to be drugs and 648 to be supplements (as defined above). Virtually every clinic participant (95.6% of the CFS; 88.6% of the ISF; and 90.3% of the *Well*) brought in a drug or supplement they had taken over the last two weeks (table 2). The average number of preparations (drugs or supplements) used was 5.8 in the CFS group (median 4, range 1–29), 4.1 in the ISF group (median 3.0, range 1–20), and 3.7 in the *Well *group (median 3, range 1–18). Overall, 85.8% of the entire sample (430 of 501) used at least one drug: 92.9% of CFS, 83.7% of ISF and 83.9% of the *Well *group. The mean number of **drugs **used per person in the CFS group was 4.3 (median 3, range 1–19); in the ISF group it was 3.0 (median 2, range 1–12), and in the *Well *group it was 2.9 (median 2, range 1–15). In contrast to drugs, the prevalence of supplement use was lower in the CFS (44.2%) and the ISF (44.3%) groups compared to the *Well *group (52.4%).

**Table 2:** Categories of medications used by subjects with chronic fatigue syndrome (CFS), insufficient symptoms/fatigue (ISF) and *Well *controls in Georgia

**Drug category**	**CFS** (n = 113)	**ISF** (n = 264)	**Well** (n = 124)	**p-value**	
	
	N (%) users			CFS vs. Well	CFS vs. ISF
Pain relievers (includes all NSAIDs and narcotics)	74 (65.5)	136 (51.5)	66 (53.2)	0.056	0.02
-NSAIDs (aspirin included)	53 (46.9)	107 (40.5)	55 (44.4)	0.74	0.31
-NSAIDs (aspirin excluded)	45 (39.8)	82 (31.1)	34 (27.4)	0.043	0.10
-Acetaminophen-containing	27 (23.9)	38 (14.4)	14 (11.3)	0.011	0.026
-Narcotic pain relievers	19 (16.8)	16 (6.1)	5 (4.0)	0.001	0.001
-Aspirin containing	15 (13.3)	35 (13.3)	27 (21.8)	0.09	1.00
Supplements/vitamins	50 (44.2)	117 (44.3)	65 (52.4)	0.158	0.68
Anti-allergy medications (anti-histamines, nasal steroids, sympathomimetics)	46 (40.7)	94 (35.6)	35 (28.2)	0.04	0.36
Asthma medications	9 (7.96)	9 (3.4)	3 (2.4)	0.097	0.065
Cold/sinus medications (anti-histamines, sympatho-mimetics, anti-cough drugs)	46 (40.7)	95 (36.0)	34 (27.4)	0.025	0.4
Anti-histamines	40 (35.4)	74 (28.0)	27 (21.8)	0.017	0.17
Antidepressants	41 (36.3)	48 (18.2)	11 (8.9)	< 0.0001	0.0007
Female hormones (birth control and HRT)^a^	28 (30.4)	49 (24.4)	26 (28.0)	0.68	0.3
- Birth control	6 (6.5)	20 (9.9)	11 (11.8%)	0.11	0.34
- Hormone replacement	19 (20.7)	28 (13.9)	13 (14%)	0.23	0.15
Gastrointestinal, acid-reducing drugs	30 (26.6)	38 (14.4)	16 (12.9)	0.0082	0.009
All cardiovascular	21 (18.6)	46 (17.4)	26 (21.0)	0.90	0.86
Sedatives (including benzodiazepines)	20 (17.7)	18 (6.8)	5 (4.0)	0.002	0.004
- Benzodiazepines only	14 (12.4)	14 (5.3)	3 (2.4)	0.003	0.027
Lipid-lowering	13 (11.5)	31 (11.7)	13 (10.5)	0.69	0.56
Thyroid hormones	12 (10.6)	11 (4.2)	8 (6. 5)	0.28	0.04
Muscle relaxants	10 (8.9)	8 (3.0)	0	< 0.001	0.002
Antibiotics	8 (7.1)	19 (7.2)	6 (4.8)	0.53	0.82
Anti-migraine	7 (6.2)	5 (1.9)	4 (3.2)	0.47	0.047
Amphetamines	5 (4.4)	7 (2.65)	2 (1.6)	0.20	0.37
Glucose-lowering	1 (0.9)	10 (3.8)	4 (3.2)	0.51	0.13
**Any category**	**108 (95.6)**	**234 (88.6)**	**112 (90.3)**	0.12	0.03

NSAID, Nonsteroid anti-inflammatory drug, HRT, hormone replacement therapy

^a ^Percentages for female hormones are calculated for n = 386 women (n = 92 CFS, n = 201 ISF, and n = 93 *Well*)

### Use of specific medication categories

Overall, in the combined sample (n = 501), the most frequently used categories were pain relievers (55.1%), supplements (43.1%), cold/sinus drugs (34.9%) and anti-allergy drugs (34.9%) (both latter groups largely represented by antihistamines – 28.1%), female hormonal drugs (26.7% of all women), antidepressants (20.0%) and anti-acid drugs (16.8%). Table 2 provides details of frequency of use by drug category for each study group. Table 3 summarizes the results of multivariate logistic regression models predicting drug and supplement use by study groups adjusted for age, BMI, income, sex, race, and geographic area (for a detailed version of this table see Additional file 1). Compared to both the *Well *controls and the ISF group, the CFS group was significantly more likely to use pain relievers (all and narcotic), antidepressants, acid-reducing gastro-intestinal medications, sedatives (largely benzodiazepines), and muscle relaxants. Compared to the *Well *(but not the ISF) group, the CFS group was also more likely to be taking non-steroid anti-inflammatory drugs, NSAIDs, (when aspirin was excluded) and anti-allergy drugs and cold/sinus (mostly anti-histamines), and less likely to be taking aspirin. In addition, compared to the ISF group, the CFS group was more likely to be taking thyroid hormone replacement and anti-migraine drugs (all p < 0.05). We further examined those drug categories that were significantly more frequently used by the CFS group and we present the results in descending order of frequency of use.

**Table 3:** Adjusted odds ratios for associations between illness status and use of specific drug categories or supplements

**Drug category**	**CFS versus Well**		**CFS versus ISF**	
	OR (95% CI)^a^	p value	OR (95% CI)	p value

Muscle relaxants	undefined	0.000	2.76 (1.02–7.43)	0.045
Sedatives	2.49 (1.47–4.21)	0.0007	3.01 (1.49–6.11)	0.002
- Benzodiazepines	2.49 (1.29–4.80)	0.006	2.70 (1.22–6.00)	0.015
Antidepressants	2.47 (1.68–3.64)	< 0.0001	2.40 (1.42–4.04)	< 0.0001
Asthma medications	1.86 (0.94–3.67)	0.074	2.47 (0.94–6.47)	0.065
Anti-histamines	1.49 (1.09–2.03)	0.013	1.53 (0.94–2.50)	0.085
Cold/sinus	1.44 (1.07–1.93)	0.015	1.29 (0.80–2.08)	0.29
Anti-migraine	1.43 (0.75–2.73)	0.28	3.44 (1.06–11.10)	0.039
Anti-allergy	1.40 (1.05–1.88)	0.024	1.32 (0.82–2.13)	0.25
Pain relievers (includes NSAIDs and narcotics)	1.33 (1.00–1.77)	0.049	1.93 (1.20–3.11)	0.007
- Narcotic pain relievers	2.24 (1.32–3.80)	0.003	3.23 (1.55–6.75)	0.002
- Acetaminophen	1.68 (1.15–2.45)	0.007	0.52 (0.29–0.91)	0.02
-NSAIDs (aspirin excluded)	1.38 (1.02–1.85)	0.03	1.54 (0.96–2.48)	0.07
-NSAIDs (aspirin included)	1.05 (0.80–1.39)	0.71	1.35 (0.85–2.15)	0.20
- Aspirin (alone)	0.68 (0.47–0.99)	0.049	0.99 (0.47–2.06)	0.97
Gastrointestinal (all acid-reducing drugs)	1.67 (1.17–2.38)	0.005	2.17 (1.24–3.80)	0.007
Thyroid hormones (all, 31/501)	1.32 (0.79–2.18)	0.28	2.60 (1.03–6.57)	0.043
Antibiotics	1.26 (0.71–2.21)	0.43	0.88 (0.36–2.15)	0.79
Supplements	0.88 (0.66–1.17)	0.37	0.98 (0.61–1.58)	0.93
Cardiovascular drugs	0.86 (0.60–1.24)	0.42	1.08 (0.58–2.03)	0.81
Glucose-lowering (insulin and oral)	0.53 (0.08–1.71)	0.46	0.23 (0.03–1.80)	0.16

^a^, CI, confidence interval. Odds ratios were adjusted for confounding factors (age, BMI, household income) and sex and geographic area, if indicated.

Note. Results are arranged in descending order of odds ratios for use of major drug categories by the CFS group vs. the *Well *group. Right justified in the first column are drugs (individual drugs or sub-categories) from the preceding major drug category above (left justified). Supplements are included in the table for completeness.

Values of the Hosmer-Lemeshow goodness of fit test ranged from 0.16 to 0.97 (values greater than >0.05 reflect good model fit, higher values reflect better fit); individual values are presented in a detailed version of this table available as an additional file.

### Pain relievers

Pain-relievers were the most commonly used drugs in all three groups and the CFS group (65.5% use) was significantly more likely than the ISF (51.5%) or the *Well *group (53.2%) to use pain relievers (including NSAIDs and narcotic medications) (tables 2 and 3). Among users of NSAIDs, bodily pain was the most frequently reported reason for use in all diagnostic groups: 62.2% of the CFS group, 47.6% of the ISF group and 52.9% of the *Well *group. Arthritis was reported as a reason significantly more frequently in the CFS group compared to the ISF group and the *Well *group (28.9%, 12.2% and 5.9%, respectively, p = 0.004 for linear trend, p = 0.01 for CFS vs. *Well*). Headache was the second most commonly reported reason for taking NSAIDs among the ISF and *Well *groups (37% and 35.3%, respectively), but the third most frequently reported reason (22.2%) in the CFS group.

The profile of NSAID use differed between persons with CFS and *Well *controls. Among persons taking NSAIDs, 49.1% of the users in the CFS group used ibuprofen compared to the 37.7% of users in the *Well *group while, conversely, acetylsalicylic acid (aspirin) was used less frequently in the CFS group (28.3%) and the ISF group (32.7%) than the *Well *group (where virtually half (49.1%) of all NSAID use was accounted for by aspirin). Similarly, overall use of aspirin was lower in the CFS and ISF groups (13.3% of all subjects in each group) compared to the *Well *group (21.8% of subjects). Thus, persons with CFS were 32% less likely than *Well *controls to be taking aspirin (OR_adj_. = 0.68, 95% CI, 0.47–0.99, p = 0.049). Of the entire *Well *group, 11.3% reported preventive use of aspirin (for "heart health/prevention") versus only 6.2% of the entire CFS group (p = 0.17) and 5.7% of the ISF group (p = 0.05). Other reported reasons for using aspirin were mainly headache or bodily pain, with similar proportions in the three groups (5.3% of CFS, 6.8% of ISF and 8% of *Well*). After excluding aspirin from the NSAID category the difference in NSAID use between the CFS group and *Well *controls was significant (p = 0.03, table 3).

Acetaminophen-containing drugs were used significantly more frequently by the CFS group (23.9%) compared to 14.4% of the ISF group and 11.3% of the *Well *controls (tables 2 and 3). The major reported reason (over 55%) in all groups was headache. However, 37% of the CFS group used such drugs also to treat bodily pain, versus only 13.2% of ISF and 7.1% of *Well *controls.

Use of anti-migraine drugs was significantly associated with CFS when compared to the ISF group (OR_adj. _= 3.44, 95% CI = 1.06, 11.10, p = 0.04) but not when compared to the *Well *controls (tables 2 and 3).

Persons with CFS were significantly more likely than *Well *controls (ORadj. = 2.24; 95% CI, 1.32–8.8) or the ISF group (OR = 3.23; 95% CI, 1.55–6.75) to use narcotic pain relievers. Users of narcotic pain relievers reported neck and back pain as the most frequent reasons (42.1% of the users in the CFS group, 37.6% in the ISF, and 40% in the *Well *group). Other reported reasons were pain in the extremities and headache/migraine. Almost half (47.4%) of the users of narcotic pain relievers in the CFS group and 18.8% of the users in the ISF group reported just pain, without specifying its localization, as a reason.

### Antihistamines

Persons with CFS were significantly more likely than *Well *controls (p = 0.013) or the ISF group (p = 0.085) to use antihistamines, which comprised the vast majority of anti-allergy and "cold/sinus" drugs (see tables 2 and 3). Major reported reasons for using anti-histamines were allergies or colds/sinus problems (80% of antihistamine users in the CFS group, 77% in the ISF group and 82.6% in the *Well *group). Using antihistamines as a sleep aid was almost twice as common in the CFS group (20.0%) and the ISF group (20.3%) compared to the *Well *group (11.1%).

### Antidepressants

A significantly higher proportion of persons with CFS (36.3%) used antidepressants compared to *Well *controls (8.9%) and persons with ISF (18.2%) (p < 0.001 for both, see tables 2 and 3). Among users of antidepressants, the most commonly reported reason was depression (64.8%, overall or 63.4% of the CFS group, 58.3% of the ISF group and 72.7% of the *Well *group). Other reported reasons included anxiety (or "nerves") in 24.3% of the CFS group, 20.9% of the ISF group, and 9.1% of the *Well *group, and sleep problems (14.6%, 4.2% and 9.1% of the CFS, ISF and *Well *groups, respectively). Using an SDS score of 50 or higher to indicate depression [11], CFS subjects had the highest SDS index scores (56.2 ± 0.9, mean ± SEM) followed by the ISF group (50.3 ± 0.5) and the *Well *controls (36.3 ± 0.4). Within each group, the mean SDS index of persons taking antidepressants was similar to the SDS index of those not taking antidepressants: CFS: 56.2 ± 1.4 vs. 56.1 ± 1.5, respectively; ISF: 50.3 ± 1.2 vs. 46.0 ± 0.7, respectively; and *Well *controls: 36.3 ± 1.4 vs. 36.5 ± 0.6, respectively. The average doses of antidepressants (expressed for each antidepressant as percent of usual adult dose as recommended by PDR) were 142.1 ± 11.1% (mean ± SEM) in the CFS group and 119.6 ± 16.7% in the *Well *group, suggesting that the higher SDS scores in persons with CFS receiving antidepressants could not be accounted for by prescription of lower doses of antidepressants than in the control group.

### Gastrointestinal drugs (simple acid reducers, H2 blockers and proton pump inhibitors)

Persons with CFS were significantly more likely than the *Well *controls (p = 0.005) or the ISF group (p = 0.007) to use acid-reducing gastrointestinal drugs (table 3). Across the groups, the major reason for anti-acid medication use was acid reflux/heartburn, which was reported by 73.4%, followed by "gas or indigestion" (15.1%). Two persons with CFS (6.7%) and 2 persons in the ISF (5.3%) reported ulcer or gastritis as a reason for use. One person with CFS reported specifically that they were taking such drugs to reduce the stomach side effects of an NSAID (etodolac). Among users of pain-relieving/anti-inflammatory drugs only, concurrent use of anti-acid drugs was significantly more common in the CFS group – 27.0% (20 of 74) than in the ISF group – 17.7% (24 of 136) or the *Well *group – 12.1% (8 of 66), p for linear trend = 0.02. Similarly, in the entire sample, concurrent use of anti-acid drugs and pain-relieving/anti-inflammatory drugs occurred significantly more frequently in the CFS group – 17.7%, (20 of 113), than in the ISF group 9.1%, (24 of 264) or the *Well *group 6.5% (8 of 124), p for linear trend = 0.005.

### Sedatives

Persons with CFS were also significantly more likely than *Well *controls (p = 0.0007) or the ISF group (p = 0.002) to use sedatives, largely accounted for by benzodiazepines (see tables 2 and 3). Reported indications were similar among users for all three groups and included: sleep problems in 42.9%, 50% and 40%, for the CFS, ISF and *Well *group, respectively, and "anxiety, stress or nerves" in 57.1%, 50% and 40%, respectively. Fewer people used imidazopyrine for sleep (n = 7 CFS, n = 6 ISF, n = 2 *Well*), while, barbiturates were only occasionally used (n = 2 CFS and n = 1 ISF) as ingredients of anti-migraine/headache drugs.

### Muscle relaxants

Subjects in the CFS group used muscle relaxants significantly more frequently (9%) than those in the ISF group (3%) or *Well *controls (0%), see tables 2 and 3.

### Hormones

Persons with CFS were significantly more likely to use **thyroid hormones **only when compared to the ISF group (OR_adj. _= 2.60, 95% CI = 1.03, 6.57, p = 0.043) but not when compared to the *Well *group (table 3). In all groups the reported reason for thyroid hormone use was "hypothyroidism" or "thyroidectomy". Concurrent use of thyroid hormone and an antidepressant occurred in six persons from the CFS group (5.3% of the whole group or 14.6% of persons with CFS who took antidepressants) and 4 from the ISF group (1.5% of the entire ISF group or 9.8% of persons with ISF who took antidepressants) but in none from the *Well *group (p-value for linear trend = 0.004, for the whole groups, p-value for linear trend = 0.22 for the subgroups on antidepressants). However, no one reported use of thyroid hormones for the purpose of augmenting the effect of antidepressants.

The overall use of **female hormone preparations **among women was similar in the CFS (30.4%) and *Well *(28%) groups (Table 2). Despite the age-matching of CFS cases and *Well *controls, birth control drugs were used less frequently by the CFS group (6.5% of females with CFS compared to 11.8% of the *Well *females and 9.9% of females with ISF) while hormone replacement use was greater among females with CFS (20.7%) than in the ISF (13.9%) or *Well *groups (14%) but these differences did not reach statistical significance.

### Other drugs and supplements

Compared to *Well *controls, CFS subjects used less frequently supplements and cardiovascular, lipid-lowering, and glucose-lowering drugs (tables 2 and 3). However, none of these differences reached statistical significance of 0.05.

## Discussion

In this cross-sectional, case-control study of CFS in Georgia we found that virtually all participants had used a drug or a supplement during the preceding two weeks (95.6% of CFS, 88.6% of ISF, and 90.3% of *Well *controls). This is higher than the average estimate of 82% for the US population in 2004 and 2006 [16]. Among the three study groups, the highest prevalence of ***drug ***use occurred in the CFS group (~93% used at least one drug), while the highest prevalence of ***supplement ***use occurred in the *Well *group (~52.4%).

Our findings confirm those from a previous study of medication use in persons with CFS from Wichita, Kansas [7]. Both studies found significantly higher usage of pain relievers, gastrointestinal drugs, antidepressants and benzodiazepines by persons with CFS compared to *Well *controls. Unlike the Wichita study, though, persons with CFS in Georgia were *not *significantly more likely than controls to use hormones and supplements but were significantly more likely than controls to use muscle relaxants and anti-allergy and cold/sinus medications. Overall, compared to persons with CFS from the Wichita study [7], a smaller proportion of persons with CFS in Georgia used pain-relievers (65.5% in Georgia vs. 87.8% in Wichita), supplements/vitamins (44.3% vs. 62.2%), antidepressants (36.3% vs. 41.1%), antibiotics (7.1% vs. 16.7%), hormones (43.4% vs. 52.5%. among women only, 11.8% among all CFS), antihypertensive drugs (17.7% vs. 21.1%), muscle relaxants (8.9% vs. 12.2%), anti-asthma medications (7.1% vs. 12.2%), glucose-lowering drugs (0.9% vs. 4.4%.). Use of other prescription drug categories such as lipid-lowering drugs (11.5% vs.12.2%) and benzodiazepines (12.4%, vs. 11.1% respectively) was similar in Georgia and Wichita (Kansas). The relatively lower usage of most prescription drug medications by persons with CFS in Georgia compared to Wichita may reflect lower seeking of, or lower access to, health care.

The more common use of pain-relievers by persons with CFS compared to those in the ISF and the *Well *groups is not surprising because joint and muscle aches belong to the symptom complex of CFS and because most pain-relievers of the NSAID group are accessible over the counter. Persons with CFS used a variety of pain relieving/anti-inflammatory drugs to treat arthritis and bodily pain, which predominated as reasons for NSAID use (in the CFS group). The significantly more common use of narcotic pain relievers by the CFS group, as compared to either the *Well *or the ISF groups, may be due to more severe pain and/or insufficient relief from conventional pain-relievers among persons with CFS. The 27% frequency of use of NSAIDs (aspirin excluded) among controls in our study appears comparable to the 32% estimated prevalence of joint pain in the general population of Georgia, or 33% for the USA [17], as not all persons with joint/muscle pain take medications all the time. The different profile of NSAIDs use by the CFS and *Well *groups (i.e., ibuprofen was most commonly used by the CFS group and aspirin was most commonly used by the *Well *group), seems to reflect different reasons for use. Overall, almost 22% of the *Well *controls used aspirin versus only 13% in the CFS and the ISF groups. Since the major reason for use of aspirin was "heart health"/prevention, it appears that more preventive use of aspirin occurred in the *Well *group. Use of acetaminophen-containing drugs in the CFS group (~24%) was higher than the estimated national average of 19%, while both the *Well *controls and the ISF group had lower usage than the national average [16].

The higher frequency of antihistamine drugs most likely reflects higher prevalence of allergies and/or cold symptoms in the CFS population. It is notable also that the antihistamine use in our control group (21.8%) was higher than the 15% antihistamine use in a control group of another U.S. study [18] and may reflect local practices and prescription patterns. In our study, about 20% of antihistamine users in the CFS and ISF groups used antihistamines as sleep aids, which was twice as much as that in the *Well *controls (11%). Use of antihistamines, which have sleepiness and drowsiness as side effects, may also be an iatrogenic contribution to the CFS symptom complex.

Use of antidepressants by *Well *controls was ~9%, mirroring the 9.2% national prevalence of depression over a 12-month period [19]. The more frequent use of psychotropic medications (antidepressants and sedatives) in the CFS group suggests that perhaps more depressed mood, anxiety and sleep disturbance are manifested by individuals fully meeting criteria for CFS. Indeed, in our study depression and anxiety were the most common psychiatric co-morbid conditions in persons with CFS [20]. Nevertheless, regardless of the more frequent use of antidepressants at higher mean dosages, persons with CFS and ISF had higher (worse) mean scores on the Zung self-rating depression scale than did *Well *controls. These results suggest that the clinical presentation of CFS, especially in subjects on antidepressants, may be related in part to untreated or treatment resistant symptoms of depression. Indeed, symptoms of fatigue in depressed patients have been found to be particularly resistant to conventional antidepressant therapy [21,22]. Moreover, depressed patients with early life stress – overrepresented in our CFS population [23], have also been shown to be less responsive to antidepressant medication [24]. Taken together, these results suggest that in some persons with CFS and depression, particularly those on antidepressants, unresolved depressive symptoms may significantly confound the diagnosis of CFS.

We were unable to find representative data for the use of acid-reducing drugs in the USA but the 12.9% use among the *Well *group was similar to the 10% overall use of anti-acids (again within last two weeks) in other parts of the developed world [25]. Half of the of users of acid-reducing drugs in the CFS group also concurrently used NSAIDs, whose major side effects are heartburn/acid reflux, gastritis, and even ulcers. At least one person from the CFS group specified that the reason for using anti-acid drugs was to counter side effects of an NSAID. Therefore, it is possible that anti-acids may have been used to treat side effects of NSAID drugs.

The 9% use of muscle relaxants in the CFS group was significantly greater not only when compared to the ISF (3%) or the *Well *group (0%) but also when compared to the national average of 1% [26]. In the national survey, half of the users of muscle relaxants took them for more than a year [26]. Because joint/muscle pain in CFS is chronic, persons in our study may also be taking muscle relaxants for extended periods of time and may experience their side effects (e.g., drowsiness, confusion, reduced alertness), which overlap with some of the CFS symptoms and may perpetuate them (i.e., iatrogenic effects of these drugs).

The approximately two-fold more common use of thyroid hormones in the CFS group compared to the ISF group deserves further study. Hypothyroidism presents a similar clinical picture to CFS; in fact, previously unrecognized hypothyroidism was the most common exclusionary condition detected during this study [8]. Autoimmune diseases are considered exclusionary for CFS as well, but were not particularly common in the study population [8]. Subjects who were successfully treated with thyroid replacement (as evidenced by TSH and T4 levels within the normal laboratory limits) were not excluded from our study. It is possible that some subjects treated with thyroid hormones may have *chemically *controlled hypothyroidism and CFS or, alternatively, they may be *chemically *euthyroid but *functionally *hypothyroid resulting in their presentation with CFS. Additional testing to address this possibility may be needed in future studies. Co-morbid depression and other psychiatric conditions were common in persons with CFS [20]. Thyroid hormones are sometimes prescribed to augment the effects of antidepressants [27] but there was no evidence for such indications in our study despite the combined use of thyroid hormone and an antidepressant by a few subjects in the CFS and the ISF group. Therefore, such use could not explain the higher frequency of thyroid hormone use by the CFS group in comparison to the ISF group.

Persons with CFS were taking, on average, approximately 6 preparations (ranging from 1 to 29 drugs and/or supplements). Polypharmacy (the use of multiple medications) raises the question of drug interactions, side effects and also the potential to use more drugs to treat symptoms that are side effects of drugs started earlier. The problem of iatrogenic symptoms is not trivial, particularly for chronic patients, as use of multiple drugs is an increasing problem [28]. The risks and consequences of polypharmacy should be a serious concern in the setting of CFS, where symptoms are chronic, treatment is largely only symptomatic, patients have about 22 doctors' visits per year [1] and may see multiple providers who independently prescribe different medications. Side effects of certain drugs may resemble symptoms of fatiguing illness. Therefore careful evaluation with respect to potential drug side effects and also drug-drug interactions is warranted for persons with CFS.

The findings from our study should be interpreted in view of its strengths and limitations. Major strengths of our study are its population-based design and the accuracy of the collected information: all drugs and supplements were brought to clinic where a research nurse viewed them and recorded the name and the dose. A limitation to consider is that reporting the reasons for drug/supplement use may not have been perfect, as subjects were not provided with a standardized list of reasons to choose from, and health literacy may have affected the accuracy of these data. Our study was cross-sectional in nature and does not allow for proper evaluation of treatment efficacy. Also, data on drug and supplement use limited to only two weeks may not be fully representative when studying a chronic, fluctuating condition such as CFS.

## Conclusion

Our findings on medication use among persons with CFS, ISF and *Well *(controls) in Georgia have significant implications for both research and practice. Researchers should take into account that subjects with CFS usually take multiple drugs and supplements and that such use may be affecting study results (therefore, adjustments for or stratification by drug use may be needed in most studies of CFS). Future studies of drug and supplement use in subjects with CFS may need to be longitudinal, to focus on periods longer than two weeks, and collect additional data such as duration of treatment and source of prescription. Such studies may need to examine whether drug use is supported by underlying diagnoses. Also, more research is needed into the efficacy of antidepressant treatment in persons with CFS and whether it is related to history of early life stress. The most important implication for practice is that health care providers need to be aware of the use of multiple drugs and supplements (polypharmacy) in persons with CFS and consider the possible iatrogenic effects – both side effects from each drug and possible drug interactions – as potential contributors to the symptoms of their patients. Provider education programs for CFS may benefit from an overview of side effects of drugs more frequently used by persons with CFS.

## Competing interests

The authors declare that they have no competing interests.

## Authors' contributions

RSB cleaned, analyzed and interpreted the data, reviewed the literature and wrote the manuscript; JSL contributed to the statistical analysis; EMM and JFJ critically reviewed the manuscript and interpreted data; WCR was instrumental in the design of the population-based study and critically reviewed the manuscript. All authors read and approved the final version of the manuscript.

## Disclaimer

The findings and views in this report are those of the authors and do not necessarily reflect the views of the funding agency.

## Supplementary Material

Additional file 1 **Detailed version of table **3** – Adjusted odds ratios for associations between illness status and use of specific drug categories or supplements**. Click here for file
