# Supplementary material for: Use of medications by people with chronic fatigue syndrome and healthy persons: a population-based study of fatiguing illness in Georgia
Source: Health Qual Life Outcomes. 2009 Jul 20;7:67. doi: 10.1186/1477-7525-7-67 (PMC2731740; doi:10.1186/1477-7525-7-67)
Supplement: Additional file 1 — Detailed version of table 3 – Adjusted odds ratios for associations between illness status and use of specific drug categories or supplements. [file 1477-7525-7-67-S1.doc]

Additional file 1: Detailed version of table 3 - Adjusted odds ratios for associations between illness status and use of specific drug categories or supplements.

| **Drug category** | **CFS versus Well** | | | **CFS versus ISF** | | |
| --- | --- | --- | --- | --- | --- | --- |
|  | OR (95% CI) ^a^ | p value | goodness  of fit test^b^ | OR (95% CI) | p value | goodness  of fit test |
| Muscle relaxants | undefined | 0.000 |  | 2.76 (1.02-7.43) | 0.045 | 0.82 |
| Sedatives | 2.49 (1.47-4.21) | 0.0007 | 0.76 | 3.01 (1.49-6.11) | 0.002 | 0.89 |
| - Benzodiazepines | 2.49 (1.29-4.80) | 0.006 | 0.80 | 2.70 (1.22-6.00) | 0.015 | 0.96 |
| Antidepressants | 2.47 (1.68-3.64) | <0.0001 |  | 2.40 (1.42-4.04) | <0.0001 |  |
| Asthma medications | 1.86 (0.94-3.67) | 0.074 |  | 2.47 (0.94-6.47) | 0.065 |  |
| Anti-histamines | 1.49 (1.09-2.03) | 0.013 | 0.97 | 1.53 (0.94-2.50) | 0.085 | 0.84 |
| Cold/sinus | 1.44 (1.07-1.93) | 0.015 | 0.84 | 1.29 (0.80-2.08) | 0.29 | 0.76 |
| Anti-migraine | 1.43 (0.75-2.73) | 0.28 | 0.77 | 3.44 (1.06-11.10) | 0.039 | 0.99 |
| Anti-allergy | 1.40 (1.05-1.88) | 0.024 | 0.72 | 1.32 (0.82-2.13) | 0.25 | 0.76 |
| Pain relievers (includes NSAID, narcotics) | 1.33 (1.00-1.77) | 0.049 | 0.42 | 1.93 (1.20-3.11) | 0.007 | 0.94 |
| - Narcotic pain relievers | 2.24 (1.32-3.80) | 0.003 | 0.31 | 3.23 (1.55-6.75) | 0.002 | 0.89 |
| - Acetaminophen | 1.68 (1.15-2.45) | 0.007 | 0.36 | 0.52 (0.29-0.91) | 0.02 | 0.71 |
| -NSAIDs (aspirin excluded) | 1.38 (1.02-1.85) | 0.03 | 0.40 | 1.54 (0.96-2.48) | 0.07 | 0.96 |
| -NSAIDs (aspirin included) | 1.05 (0.80-1.39) | 0.71 | 0.23 | 1.35 (0.85-2.15) | 0.20 | 0.61 |
| - Aspirin (alone) | 0.68 (0.47-0.99) | 0.049 | 0.75 | 0.99 (0.47-2.06) | 0.97 | 0.66 |
| Gastrointestinal (all acid-reducing drugs) | 1.67 (1.17-2.38) | 0.005 | 0.52 | 2.17 (1.24-3.80) | 0.007 | 0.72 |
| Thyroid hormones (all, 31/501) | 1.32 (0.79-2.18) | 0.28 | 0.50 | 2.60 (1.03-6.57) | 0.043 | 0.76 |
| Antibiotics | 1.26 (0.71-2.21) | 0.43 | 0.66 | 0.88 (0.36-2.15) | 0.79 | 0.69 |
| Supplements | 0.88 (0.66-1.17) | 0.37 | 0.69 | 0.98 (0.61-1.58) | 0.93 | 0.13 |
| Cardiovascular drugs | 0.86 (0.60-1.24) | 0.42 | 0.68 | 1.08 (0.58-2.03) | 0.81 | 0.84 |
| Glucose-lowering (insulin and oral) | 0.53 (0.08-1.71) | 0.46 |  | 0.23 (0.03-1.80) | 0.16 | 0.69 |

Note. Results are arranged in descending order of odds ratios for use of major drug categories by the CFS group vs. the *Well* group. Right justified in the first column are drugs (individual drugs or sub-categories) from the preceding major drug category above (left justified). Supplements are included in the table for completeness.

^a^, CI, confidence interval. Odds ratios were adjusted for confounding factors (age, BMI, household income) and sex and geographic area, if indicated.

^b^, H-L, Hosmer-Lemeshow goodness of fit test; p values >0.05 show a good model fit, higher p values reflect better model fit.
